# Characteristics of recent HIV infection among individuals newly diagnosed as HIV-positive in South Korea (2008–2015)

**DOI:** 10.1038/s41598-022-13953-0

**Published:** 2022-06-22

**Authors:** Myeongsu Yoo, Jin-Sook Wang, Su-Jin Park, Jeong-ok Cha, Yoonhee Jung, Yoon-Seok Chung, Myung Guk Han, Byeong-Sun Choi, Sung-Soon Kim, Mee-Kyung Kee

**Affiliations:** 1 Division of Clinical Research, Center for Emerging Virus Research, Korea National Institute of Health, Korea Disease Control and Prevention Agency, Chungbuk, Republic of Korea; 2 Division of Public Health Emergency Response Research, Director General for Public Health Emergency Preparedness, Korea Disease Control and Prevention Agency, Chungbuk, Republic of Korea; 3 Division of Viral Disease, Bureau of Infectious Disease Diagnosis Control, Korea Disease Control and Prevention Agency, Chungbuk, Republic of Korea; 4 Division of TB and HIV/AIDS Control, Center for Disease Prevention, Korea Centers for Disease Control & Prevention, Chungbuk, Republic of Korea; 5 Central Disease Control Headquarters, Korea Disease Control and Prevention Agency, Chungbuk, Republic of Korea; 6 Division of HIV/AIDS Prevention and Control, Bureau of Infectious Disease Policy, Korea Disease Control and Prevention Agency, Chungbuk, Republic of Korea; 7 Honam Regional Center for Disease Control and Prevention, Regional Centers for Disease Control and Prevention, Korea Disease Control and Prevention Agency, Gwangju, Republic of Korea; 8 Division of Viral Disease Research, Center for Infectious Diseases, Korea National Institute of Health, Chungbuk, Republic of Korea; 9 Division of Chronic Viral Diseases, Center for Emerging Virus Research, Korea National Institute of Health, Korea Disease Control and Prevention Agency, Chungbuk, Republic of Korea; 10 Center for Infectious Diseases Research, Korea National Institute of Health, Chungbuk, Republic of Korea; 11 Center for Vaccine Research, Korea National Institute of Health, Korea Disease Control and Prevention Agency, Chungbuk, Republic of Korea; 12 International Tuberculosis Research Center, Seoul, Republic of Korea

**Keywords:** Diseases, Health care

## Abstract

Most HIV-positive individuals diagnosed in Korea are infected through sexual contact, with the time of HIV infection therefore often being unknown. The aim of this study was to investigate the characteristics of diagnosed patients near the time of HIV seroconversion to establish useful HIV intervention strategies. Cross-sectional study. Newly diagnosed HIV cases based on the national HIV surveillance system in South Korea, 2008–2015. To distinguish recent from long-standing HIV infection among 5898 nationwide HIV-positive specimens, limiting antigen avidity assays (Sedia HIV-1 LAg-Avidity EIA, cut-off = 1.5) were performed. Data on CD4+ T cell count at HIV diagnosis and death from AIDS within one year after first HIV diagnosis were used to distinguish recent HIV infections. Acute HIV infection is characterized by detectable HIV RNA or HIV p24 antigen levels, along with a negative or indeterminate antibody western blot result, but with the subject subsequently testing positive after a predetermined period. We analyzed the characteristics of recent and acute HIV infections by sex, age, nationality, HIV testing site, region, and reason for HIV testing. Recent and acute HIV infections were described as the proportion of recent and acute HIV infections among newly-diagnosed cases in a given year. Recent and acute HIV infections over the 8-year study period were 20.5% (1210/5898) and 9.5% (562/5898), respectively. Both infections were generally higher in the following groups: males, younger age, Koreans, individuals who were tested due to disease, residents of smaller city or rural area, and individuals diagnosed in recent calendar years. Acute infections were significantly higher among individuals tested in hospitals and due to suspected HIV infection. The recent and acute HIV infection in younger age groups (< 30 years) has been increasing in a given year. There was an increase in the proportion of young individuals with recent HIV infection, and we identified risk groups more at risk of HIV infection recently in Korea. Therefore, our results could prove useful for the development and assessment of national HIV prevention strategies in Korea and provide valuable data for further HIV research.

## Introduction

The number of people worldwide living with the human immunodeficiency virus (HIV) was estimated at 37.7 million and acquired immunodeficiency disease syndrome (AIDS)-related deaths at 680,000 in 2020. The development of treatments and prevention strategies against HIV infection led to new HIV infections declining from the peak of 3.4 million in 1996 to 1.5 million in 2020. Accordingly, the Joint United Nations Programme on HIV/AIDS (UNAIDS) had planned the “95–95–95” targets for the end of the HIV epidemic worldwide by 2030^1^. However, these targets require optimizing strategies for the earliest possible diagnosis of HIV infection. In Korea, the first case of HIV infection was detected in 1985; as of 2018, the cumulative number of individuals infected with HIV was 17,502, and overall HIV prevalence was estimated to be less than 0.1%. Korea was considered a low HIV prevalence country, but new cases of HIV infection have rapidly increased since the early 2000’s, with the number being 244 in 2000, 837 in 2010, and 1016 in 2020^2,3^.

A primary goal of the national HIV/AIDS program in Korea is to reduce new HIV infection. This is measured based on HIV incidence, described as the rate of individuals newly infected with HIV in a population. As HIV incidence is an essential indicator for monitoring HIV/AIDS epidemic trends, it is used to evaluate the effectiveness of HIV prevention and treatment programs; in keeping with this worldwide trend, national HIV surveillance indicators have shifted from HIV prevalence to HIV incidence^4^. Several serological tests have been developed for distinguishing between recent and long-term HIV infection^5,6^. In the United States (US), a limiting antigen (LAg) avidity enzyme immunoassay was developed by the Centers for Disease Control and Prevention (CDC) for cross-sectional HIV incidence estimation. This assay measures the avidity of antibody binding to a multi-subtype protein derived from an immune-dominant region of glycoprotein 41^7^. The CDC developed the serological testing algorithm for estimating recent HIV infection in 1998. Currently, the incidence and prevalence are reported according to epidemiological factors including age, race, and sexual behaviors using the CD4+ model and the Bayesian hierarchical model^8^. In the United Kingdom (UK), Public Health England has estimated the incidence of HIV since the establishment of a nationwide HIV surveillance project in 2008. Through this project, nationwide HIV incidence in high-risk groups, such as homosexual and bisexual men, has been monitored and reported^9^.

We established a protocol for using testing based on the HIV LAg Avidity assay to identify recent HIV infections among HIV-positive individuals^10^ and have tested HIV-positive individuals for recent HIV infection each year since 2008. Approximately 1000 newly-diagnosed cases of HIV infection have been reported continuously each year since 2011 in Korea. Therefore, it is necessary to identify the characteristics of individuals recently infected with HIV and to establish prevention strategies against HIV infection for target transmission groups. In 2015, the Korea Disease Control and Prevention Agency (KDCA, former the Korea Centers for Disease Control and Prevention) established a surveillance system to monitor recent HIV infections as part of the national HIV surveillance system. In the current study, we aimed to identify the characteristics of recent HIV infections among newly-diagnosed cases in Korea from 2008 to 2015. We investigate the characteristics of patients near the time of seroconversion, in order to establish useful HIV intervention strategies. Therefore, we endeavor to improve the national HIV laboratory surveillance system by assessing and critically evaluate the newly implemented surveillance system.

## Material and methods/experimental details/methodology

### Korea national HIV laboratory surveillance system: recent HIV infection surveillance system

In Korea, enzyme-linked immunosorbent assay (ELISA) or rapid tests are used for HIV screening at primary testing sites, including public health centers (PHCs), hospitals, clinics, and blood centers. When a specimen tests positive at a primary testing site, further testing of the specimen is requested at a secondary testing institute—the regional Institutes of Health and Environment (RIHE)—where western blotting (WB) is used in addition to HIV screening tests to confirm HIV infection. Until 2005, all specimens with positive or indeterminate WB results were sent to the KDCA for final confirmatory testing of HIV. Since 2006, the process of HIV testing has been reduced from three stages to two to reduce turnaround time between screening and confirmatory testing^3^, and 17 RIHE are allowed to make the final confirmatory diagnoses. However, a few specimens that result in indeterminable diagnoses at the RIHE are sent to the KDCA. In 2013, the KDCA launched a surveillance system to monitor recent HIV infection as part of the national HIV surveillance system to establish effective national-level HIV infection prevention policies and calculate the national statistical indices for HIV. To establish this new surveillance system to monitor recent HIV infection, most HIV-positive specimens submitted to and stored at the RIHE between 2006 and 2014 were collected. Since 2015, the KDCA has collected HIV-positive specimens annually from the 17 RIHE. To distinguish between recent and long-standing HIV infection among the collected HIV-positive specimens, LAg testing was performed.

### Specimens

The KDCA not only collects HIV-positive specimens from the RIHE, but also from the HIV/AIDS Supporting Network System (HASNet). The latter is the central database for the management of patients infected with HIV, from testing sites and PHCs nationwide. Data that is collected from HIV-positive specimens includes: date of diagnosis, date of birth, mode of transmission, the reason for HIV testing, marital status, HIV screening facility, CD4+ cell count, and viral load. A total of 10,423 HIV-positive specimens collected between 2008 and 2015, along with their necessary data, were used for use this study. All these specimens tested positive using particle agglutination, ELISA, and WB tests. From these specimens, those from registered individuals infected with HIV who had been previously confirmed to be HIV-positive were excluded. Multiple specimens obtained from the same individual were also excluded—only the first specimen from each individual was included for this study. Lastly, anonymous specimens that could not be checked for potential duplication were excluded.

**Figure 1:**
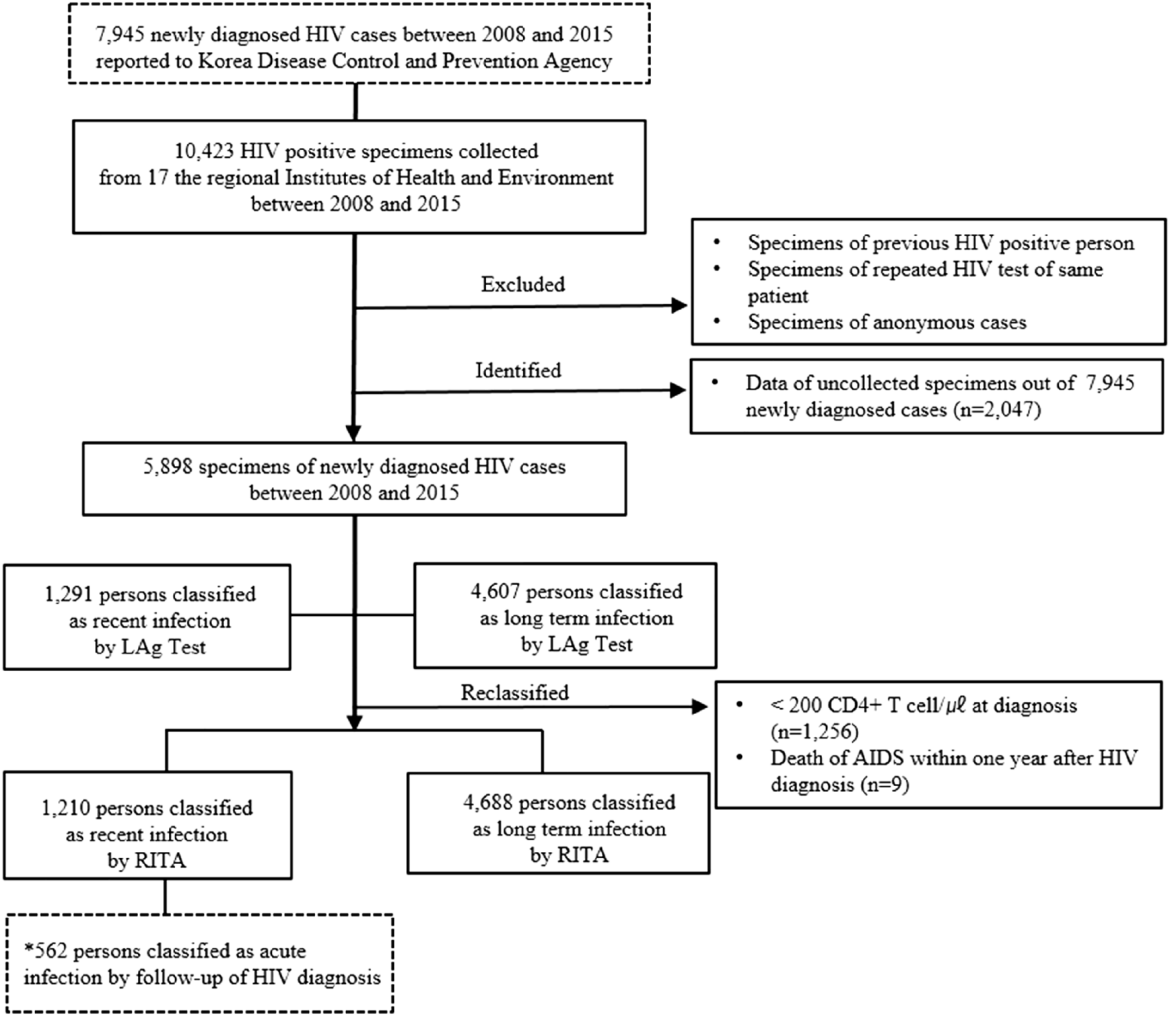
Flowchart for classification as recent and acute HIV infection among HIV-positive specimens from diagnosed infection cases in Korea between 2008 and 2015. Asterisk: 562 persons were identified prior to confirm HIV seroconversion. *RITA* resent infection testing algorithm.

According to the natural history of HIV, HIV attacks the body's immune system and leads to a gradual decline in the number of CD4+ T cells, a key immune marker, leading to the development of AIDS. The US CDC classified the occurrence of AIDS once the CD4+ T cell count drops below 200 cells/mm^3^^11^. In accordance with the WHO’s recommendation to consider clinical results, the recent infection testing algorithm (RITA) is employed to minimize the false recent ratio (FRR) of LAg-Avidity assays^12,13^. Among the 5898 specimens finally included in this study, CD4+ T cell testing at HIV diagnosis had been performed in 2972 cases—1256 individuals had a CD4+ T cell count < 200 at HIV diagnosis. Nine individuals without CD4+ T cell counts died within 1 year due to AIDS after HIV diagnosis. Eighty-one cases categorized as recent HIV infections based on the LAg Avidity assay were reclassified as long-term HIV infections (Fig. 1). This study was obtained approval for study design from the KDCA Institutional Review Board (approval no. 2016-07-02-PE-A). Also, the informed consents from study participants were waived the requirement for written by the KDCA Institutional Review Board.

### Laboratory testing

The LAg Avidity assay (Sedia HIV-1 LAg-Avidity EIA; Sedia Biosciences Corporation, Portland, OR, USA) is a single-well avidity enzyme immunoassay. The antigens used was a multi-subtype recombinant protein containing the major variants of glycoprotein 41 immunodominant regions from the HIV-1 group M viruses^14,15^. A specific buffer (pH 3.0) was used as the dissociation agent to remove low avidity HIV-1 antibody. Avidity was reported as the normalized optical density (ODn). We tested 5895 specimens according to manufacturer’s kit insert. According to the manufacturer’s instructions, the estimated mean duration of recent HIV infection (MDRI), at a cut-off for ODn of 1.5, is 130 days (95% confidence interval [CI]: 118–142)^16^. The recent HIV infection period encompasses the acute HIV infection period.

From the 1210 specimens tested as recent infections, a total of 562 specimens were classified as acute HIV infection. This means that patients presented at the phase of HIV infection that occurs immediately after HIV infection. Acute HIV infection is characterized by detectable HIV RNA or HIV p24 antigen and a negative or indeterminate result in an antibody WB test but with the patient subsequently testing positive after a certain period of time according to the KDCA's laboratory HIV diagnosis protocol. The subsequent WB test will be deemed positive when more than three bands in total occur, more than two bands among gp120, gp160, and gp41 occur, and more than one band among p24 or p31 occur. Acute HIV infection covers Fiebig stages I through V. We consider the mean duration of acute HIV infection to be 65 days, including 30 days for Fiebig stages I to IV and 35 days for the first half of Fiebig stage V^17^.

### Statistical analysis

We used a multi-assay algorithm, which combines data from assays and considers CD4+ T cell counts at HIV diagnosis and AIDS-related death within 1 year after HIV diagnosis, to determine recent HIV infection. Accordingly, recent HIV infection was described as the proportion of recent HIV infections among newly-diagnosed cases of HIV infection in a given year. Acute HIV infection was described as the proportion of acute HIV infections among newly-diagnosed HIV cases in 1 year. We compared and analyzed HIV testing site (hospitals, PHCs, blood centers), region (metropolis, smaller city or rural), and reason for HIV testing in a longitudinal cross-sectional manner. The reasons for HIV testing were divided into the following five categories: health check-up, due to disease (general medical examination, hospitalization, diseases including the suspicion of HIV itself by physician, or the presence of typical opportunistic infections), due to suspected infection (voluntary test), preoperative testing, others (including perinatal check-up, blood donation, partner of HIV-positive individual, and sexually transmitted infection risk group), and unknown. Annual trends of proportion of recent (or acute) infections were analyzed using the chi-square (trend) test. The odds ratios for recent and acute HIV infections were estimated using logistic regression analysis. All analyses were performed using SAS 9.4; statistical significance was set at 0.05 for all cases.

### Ethical approval

All authors hereby declare that all experiments have been examined and approved by the appropriate ethics committee (the KDCA Institutional Review Board Ethics Committee; approval number: 2016-07-02-PE-A), and have therefore been performed in accordance with the ethical standards laid down in the 1964 Declaration of Helsinki.

### Consent

The requirement for informed consent from study participants was waived by the KDCA Institutional Review Board.

## Results

### Status of HIV infection and characteristics of recent HIV infection in Korea (2008–2015)

The number of specimens from newly identified HIV-positive individuals over the 8-year period between 2008 and 2015 collected to assess recent HIV infection was 5898, accounting for 74.2% of 7945 newly identified HIV-positive individuals (Table 1). Of 5898 cases, 486 (8.2%) were non-Koreans. The male-to-female ratio was 10.9:1 (5402:496). The high proportions were observed in the 20–29 years age group (25.1%) and in the 30–39 years (25.2%), followed by 40–49 years (23.0%), and 50–59 years (15.7%) age groups. Based on the reason for HIV testing, besides the others group, approximately 28.8% of new cases were detected by testing of cause for disease.

**Table 1:** The characteristics of study population for the recent HIV infection study in Korea from 2008 to 2015.

Category	Study population
	N (%)
Total	5898 (74.2)*
**Sex**	
Male	5402 (91.6)
Female	496 (8.4)
**Age, years**	
< 20	177 (3.0)
20–29	1482 (25.1)
30–39	1485 (25.2)
40–49	1355 (23.0)
50–59	926 (15.7)
60 ≤	473 (8.0)
**Nationality**	
Koreans	5412 (91.8)
Non-Koreans	486 (8.2)
**Testing site**	
Hospitals	4644 (78.7)
Public health centers	1139 (19.3)
Blood center	115 (2.0)
**Region**	
Metro	4180 (70.9)
Small	1718 (29.1)
**Reason for testing**	
Health check-up	700 (11.9)
Due to disease	1701 (28.8)
Suspected	838 (14.2)
Preoperative test	777 (13.2)
Others	1882 (31.9)
**Year**	
2008	391 (6.6)
2009	502 (8.5)
2010	648 (11.0)
2011	715 (12.1)
2012	761 (12.9)
2013	891 (15.1)
2014	993 (16.9)
2015	997 (16.9)

*Proportion of specimens for recent HIV testing among 7945 newly HIV diagnosed individuals in Korea from 2008 to 2015.

*Metro* metropolis, *small* smaller city or rural area, *others* prenatal check-up, blood donation, partner of HIV positive, sexual transmitted infection risk group, etc. Age median of specimens for study: 38 (28–49).

Recent HIV infection and acute HIV infection in these individuals during this 8-year period was 20.5% and 9.5%, respectively (Table 2). Recent HIV infection was significantly higher in male (21.1%) than in female (14.1%, adjusted odds ratio [AOR]: 1.35, 95% CI: 1.03–1.78). Recent HIV infection was approximately four times higher in the < 20 years (35.0%; AOR: 4.48, 95% CI: 2.91–6.91) and 20–29 years (33.9%; AOR: 4.13, 95% CI: 3.02–5.64) age groups than in the ≥ 60 years age group. Recent HIV infection was about two times higher in the Koreans (21.1%; AOR: 1.56, 95% CI: 1.17–2.08) than in non-Koreans (p < 0.001). Regarding the reason for testing, recent HIV infection was highest in those tested for HIV due to disease (25.6%; AOR: 1.78, 95% CI: 1.49–2.12).

**Table 2:** The logistic regression analysis of characteristics of the recent HIV infection including acute HIV infection in Korea from 2008 to 2015.

Category	Recent HIV infection			Acute HIV infection		
	N (%)*	Single logistic regression, OR (95% C.I.)	Multiple logistic regression, AOR (95% C.I.)	N (%)^§^	Single logistic regression, OR (95% C.I.)	Multiple logistic regression, AOR (95% C.I.)
Total	1210 (20.5)			562 (9.5)		
**Sex**						
Male	1140 (21.1)	1.63 (1.25–2.11)	1.35 (1.03–1.78)	539 (10.0)	2.28 (1.49–3.50)	1.67 (1.07–2.62)
Female	70 (14.1)	1	1	23 ( 4.6)	1	1
**Age, years**						
< 20	62 (35.0)	4.18 (2.75–6.36)	4.48 (2.91–6.91)	16 ( 9.0)	1.94 (1.00–3.77)	2.25 (1.13–4.50)
20–29	503 (33.9)	3.99 (2.94–5.40)	4.13 (3.02–5.64)	234 (15.8)	3.67 (2.36–5.71)	4.45 (2.82–7.03)
30–39	279 (18.8)	1.80 (1.31–2.45)	1.82 (1.32–2.49)	142 ( 9.6)	2.07 (1.32–3.26)	2.12 (1.33–3.38)
40–49	192 (14.2)	1.28 (0.93–1.77)	1.22 (0.88–1.69)	89 ( 6.6)	1.38 (0.86–2.20)	1.26 (0.78–2.03)
50–59	120 (13.0)	1.16 (0.82–1.63)	1.11 (0.79–1.57)	58 ( 6.3)	1.31 (0.80–2.15)	1.17 (0.70–1.94)
60 ≤	54 (11.4)	1	1	23 ( 4.9)	1	1
**Nationality**						
Koreans	1,144 (21.1)	1.71 (1.31–2.23)	1.56 (1.17–2.08)	552 (10.2)	5.41 (2.87–10.17)	3.66 (1.89–7.06)
Non-Koreans	66 (13.6)	1	1	10 ( 2.1)	1	1
**Testing site**						
Hospitals	918 (19.8)	1	1	485 (10.4)	1	1
PHCs	259 (22.7)	1.20 (1.02–1.38)	1.02 (0.85–1.24)	75 ( 6.6)	0.60 (0.47–0.78)	0.50 (0.37–0.68)
Blood center	33 (28.7)	1.64 (1.08–2.46)	1.00 (0.65–1.54)	2 ( 1.7)	0.15 (0.04–0.62)	0.16 (0.04–0.66)
**Region**						
Metro	770 (18.4)	1	1	300 (7.2)	1	1
Small	440 (25.6)	1.40 (1.22–1.60)	1.61 (1.40–1.85)	262 (15.3)	1.96 (1.64–2.34)	2.45 (2.04–2.95)
**Reason for testing**						
Health check-up	139 (19.9)	1	1	48 (6.9)	1	1
Due to disease	435 (25.6)	1.39 (1.12–1.72)	1.79 (1.50–2.14)	299 (17.6)	2.90 (2.11–3.98)	2.89 (2.06–4.04)
Suspected	199 (23.7)	1.26 (0.98–1.65)	1.24 (0.98–1.56)	86 (10.3)	1.55 (1.08–2.25)	1.40 (1.13–2.56)
Preoperative test	134 (17.2)	0.84 (0.65–1.09)	0.96 (0.73–1.27)	68 (8.8)	1.30 (0.89–1.94)	1.19 (0.80–1.78)
Others	303 (16.1)	0.78 (0.62–0.97)	0.97 (0.78–1.23)	61 (3.2)	0.46 (0.31–0.67)	0.56 (0.38–0.84)

*Proportion of recent HIV infection among specimen for recent HIV testing.

^§^Proportion of acute HIV infection among specimen for recent HIV testing.

*PHCs* public health centers, *metro* metropolis, *small* smaller city or rural area, *others* prenatal check-up, blood donation, partner of HIV positive, sexual transmitted infection risk group, etc., *OR* odds ratio, *AOR* adjusted (sex, age, nationality, testing site, region, reason for testing) odds ratio.

Age median (IQR, Q1–Q3) of individuals with recent HIV infection: 31 (24–43), age median of individuals with acute HIV infection: 32 (25–42).

Acute HIV infection was significantly higher in male than in female (AOR: 1.67, 95% CI: 1.07–2.62), highest in the 20–29 years age group (15.8%; AOR: 4.45, 95% CI: 2.82–7.03), and about four times higher in Koreans than in non-Koreans (AOR: 3.66, 95% CI: 1.89–7.06). Acute HIV infection was highest (17.6%; AOR: 5.09, 95% CI: 3.79–6.83) in those tested for HIV due to disease. Acute HIV infections in the suspected disease, preoperative testing, and health check-up groups were significantly higher than that in the others group (Table 2).

### Characteristics of trends in recent and acute HIV infection

**Figure 2:**
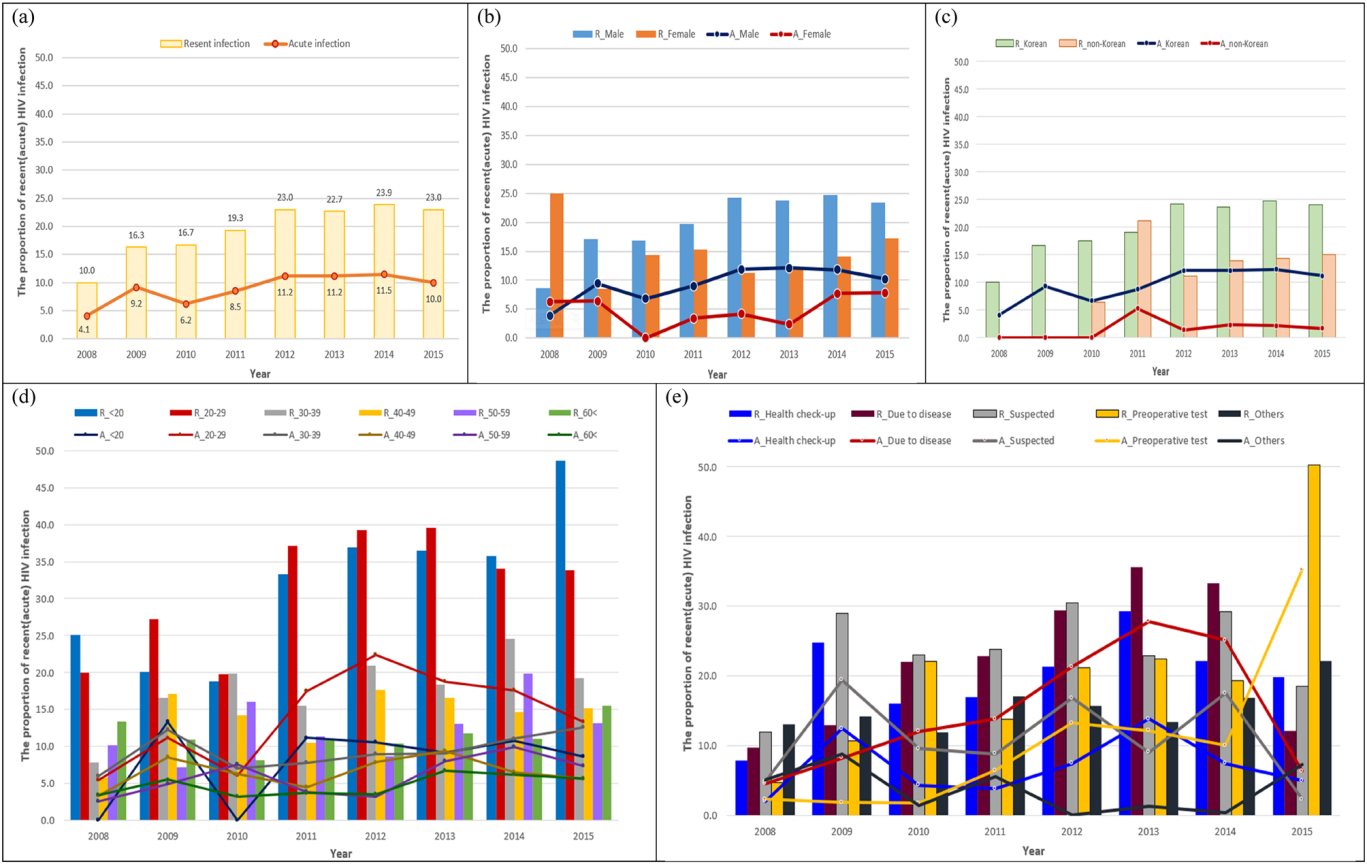
Comparison of recent and acute HIV infection rates by gender, nationality, age group, reason for HIV testing. (**a**) Recent and acute HIV infection rate. (**b**) Recent and acute HIV infection rate by gender. (**c**) Recent and acute HIV infection rate by nationality. (**d**) Recent and acute HIV infection rate by age group. (**e**) Recent and acute HIV infection rate by reason for testing. *R*: recent infection, *A*: acute infection.

We performed a comparative analysis of the trends in recent and acute HIV infections in Korea by variables between 2008 and 2015 (Fig. 2). Both annual recent and acute HIV infections were found to show increasing trends *(*P < 0.001). Recent HIV infection was 10.0% in 2008 but increased to 19.3% in 2011 and was consistently > 22% between 2012 and 2015 (P < 0.001). Moreover, acute HIV infection increased from 4.1% in 2008 to ≥ 10.0% since 2012 *(*P < 0.001) (Fig. 2a). Both annual recent and acute HIV infections were higher in male, respectively (P < 0.001, P = 0.004) (Fig. 2b). Annual recent HIV infection showed an increasing trend in younger age groups—significant changes in recent HIV infection were noted in the < 20 years and 20–29 years age groups (P < 0.001). In the < 20 years age group, recent HIV infection showed a striking increase from 18.8% in 2010 to 48.6% in 2015 *(*P < 0.001). In the 20–29 years age group, it increased from 20.0% in 2008 to 39.6% in 2013, but reduced slightly to approximately 34% in 2014 and 2015. Acute HIV infection was higher in the 20–29 years group than in the other age groups between 2011 and 2015, particularly in 2012, when it was 22.4% (Fig. 2d). Annual recent HIV infection was generally lower in non-Koreans than in the Koreans (P = 0.04). Similarly, annual acute HIV infection was lower in non-Koreans than in the Koreans (Fig. 2c). For HIV test takers due to disease, both annual recent and acute HIV infection were overall higher than in other groups (P < 0.001), and showed increasing trends (P < 0.001). The group that underwent HIV testing with suspected HIV infection showed the highest recent HIV infections from 2009 to 2012. Those diagnosed for HIV infection by preoperative testing showed increasing trends in annual recent HIV (P = 0.04) as well as annual acute HIV infections (P = 0.03). In 2015, they had the highest recent and acute HIV infections. (Fig. 2e).

### Characteristics of recent and acute HIV infection in younger age groups

**Figure 3:**
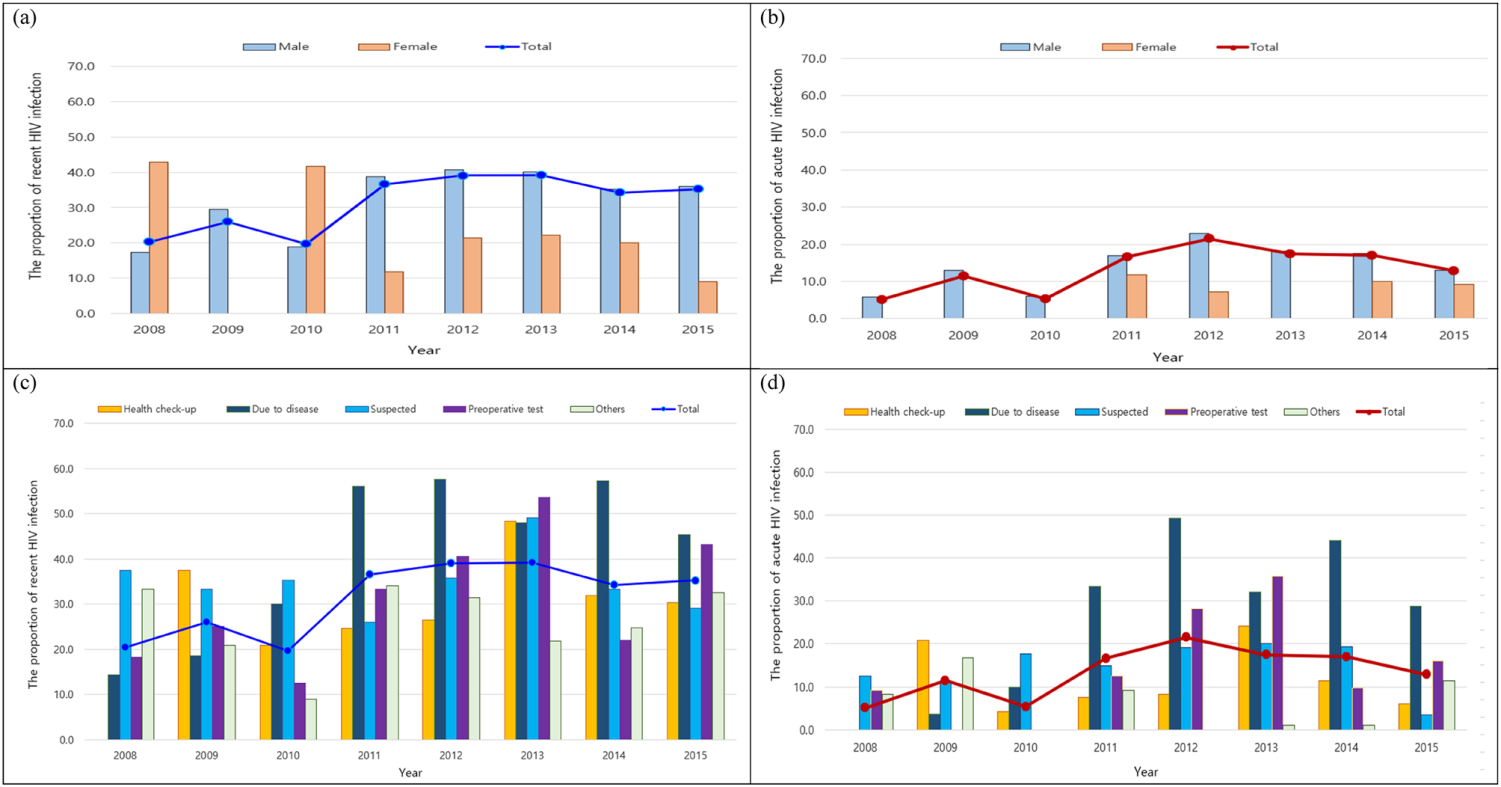
Comparison of recent and acute HIV infection rates by gender, reason for HIV testing under 30 years of age. (**a**) Recent HIV infection rate under 30 age group by gender. (**b**) Acute HIV infection rate under 30 age group by gender. (**c**) Recent HIV infection rate under 30 age group by reason for HIV testing. (**d**) Acute HIV infection rate under 30 age group by reason for HIV testing.

For younger age groups (< 20 years and 20–29 years) between 2008 and 2015, we investigated the characteristics of annual recent and acute HIV infection (Fig. 3). The recent HIV infection in younger age groups increased annually during study period (P = 0.0005). Those increased from approximately 20% in 2008 to approximately 40% in 2012 and decreased to about 35% in 2014 and 2015. Acute HIV infection showed an increasing trend during 8 years (P = 0.004), those showed reaching approximately 20% in 2012, and then a decreasing trend from 2013. The annual recent HIV infection was higher in male than in female, except in 2008 and 2010 (P = 0.0027) (Fig. 3a). The annual acute HIV infection was higher in male than in female during the study period (Fig. 3b). The annual recent HIV infection the among test takers due to disease increased (P = 0.04) and were the highest from 2011 to 2015, except in 2013. For annual recent HIV infection, the patients detected by preoperative testing showed an increasing trend (P = 0.01) and the suspected showed over 20% during the study period and increased to about 50% in 2013 (Fig. 3c). For acute HIV infection by reason for HIV testing, the test takers due to disease were showed an increasing trends (P = 0.02). The test takers due to disease were the highest from 2011 to 2015, and those due to suspected disease were 10–20% during the study period, except in 2015 (Fig. 3d).

## Discussion

Surveillance systems for both recent and acute HIV infections allow for an understanding of HIV infection status within 130 and 65 days of HIV infection, respectively. The outcome of the current study may provide a good index to evaluate the current HIV diagnosis system in Korea and provide useful evidence for additional prevention policies against HIV infection. Recent and acute HIV infection over an 8-year period (between 2008 and 2015) was 20.5% and 9.5%, respectively, and was generally higher observed in the following groups: male, younger age, Koreans, residents at smaller city or rural area, individuals who were tested due to disease, and more recently detected HIV cases. Recent HIV infection was the highest in men aged < 20 years and 20–29 years, and recent HIV infections have been increasing every year. However, the proportion of acute HIV infection was about half of recent HIV infection, and annual changes have mimicked the trend observed for recent HIV infection. Furthermore, we found that the time of testing for HIV infection was later for those < 20 than for those aged 20–29 years. In Korea, it has been reported that most of adolescents (< 20) have few opportunities for HIV-related education, such as HIV-related diseases, infection routes, and test education^18^. To enforce increased access to HIV testing among adolescents, we suggested that education should be increased on where and how to take an HIV test, the possible routes of HIV infection, and prevention of infection. Both recent and acute HIV infection in non-Koreans were lower than those in Koreans, which may indicate that HIV testing was less accessible to foreigners than to Koreans. To prevent the increase in the number of non-Koreans with HIV in Korea, it is necessary to develop a plan to promote HIV screening for non-Koreans.

The cumulative number of individuals infected with HIV over the 23-year period between 1985 and 2007 was 5964; the male-to-female ratio was 8.4:1 and 12.1% of the entire group comprised foreigners^3^. Compared to this pre-2008 data, the number of individuals infected with HIV identified during the 8-year period between 2008 and 2016 increased by nearly 2000 (7945 in total). In addition, the number of infected males was more compared to that shown by the pre-2008 data. Moreover, the annual number of new cases was below 1000 between 2008 and 2012, but has increased to more than 1100 since 2013. The age at first detection of HIV infection has also decreased, and the proportion of individuals diagnosed with HIV has recently increased in individuals in their 20’s than in the other age groups.

Acute HIV infection in Korea was first identified in 1999 and accounted for only 0.5% of all individuals infected with HIV identified that year^19^. Since then, the proportion of acute HIV infections among newly identified individuals infected with HIV has been increasing every year. Prior to the establishment of the surveillance system to monitor recent HIV infections in 2013, the 4^th^ generation HIV testing kit was commonly used to detect early HIV infection at screening sites. Consequently, the detection of acute HIV infection increased during the screening process. General characteristics of acute HIV infection include a high male-to-female ratio of 18:1 (higher than the proportion in the entire group of newly identified individuals infected with HIV) and younger age at the time of detection^19^. These findings have allowed for the prediction of changes in the characteristics of newly identified individuals infected with HIV, and the results of prediction are reflected in the current characteristics of HIV infection.

Although no cure or vaccine for HIV is available, antiviral medications to suppress viral replication have allowed for the prevention of person-to-person transmission and progression to AIDS. However, individuals infected with HIV need to be on medications for the rest of their lives. Consequently, they may develop drug resistance, experience adverse effects, and face social stigma directed against individuals infected with HIV. Furthermore, the need for lifelong medication will increase the socioeconomic burden on both individuals and their country. In Korea, a national-level surveillance system has been established as a first step to prevent the spread of HIV infection, which allows for early detection of individuals infected with HIV and shortens the time required for HIV testing. First, the policy to allow for early detection of individuals infected with HIV increases access to HIV testing, so that individuals in the general population can determine their HIV infection status. Since 2014, self-diagnosis using OraQuick—a type of rapid testing kit—has been permitted. In addition, free HIV testing has been made available at PHCs, and an anonymous HIV testing service is available at both general hospitals and PHCs^20^. Second, for early detection of HIV infection, it was necessary to shorten the time between the HIV screening test and the final diagnosis of HIV infection. Consequently, the three-stage system (screening sites, RIHE, KDCA) was changed to a two-stage system (screening sites, RIHE), which shortened the duration of the HIV testing process by 10 days^21^. In 2015, an algorithm to confirm the diagnosis of early-stage infection was implemented to further reduce the time required for diagnosis^21^. With these efforts, early detection of individuals infected with HIV has become possible, but the number of new HIV cases has increased.

Previously, the US CDC developed a BED capture enzyme immunoassay to estimate HIV incidence at the population level^22^, but a high FRR led to overestimation of HIV incidence. To improve the accuracy of the assay, they developed a new avidity assay, the HIV-1 LAg-Avidity EIA. This improved assay has resulted in low FRR in some studies due to the use of multi-subtype antigens and use of avidity biomarker. It seemed to be less affected by low CD4+ T cell counts or disease states than the BED assay^23^. In 2008, the World Health Organization (WHO) organized a technical working group on HIV incidence to develop guidelines for HIV incidence assays and recent HIV infection testing algorithms. In every annual meeting, updates on methods for MDRI and FRR estimation were major issues^12,13^. In our neighboring countries, there have been studies on recent HIV infection during a similar period. From 2006 to 2015, recent HIV infections among newly-diagnosed cases in three metropolitan areas in Japan, Tokyo, Osaka, and Fukuoka, were 38.6%, 30.1%, and 20.4%, respectively^24^. In the nine-year survey period from 2008 to 2016, the proportion of recent infections among men who have sex with men (MSM) in Beijing, China was 40.6% (106/261), although this value would have been overestimated due to the application of the BED assay instead of the LAg assay^25^. Recent HIV infections in metropolises, including in Seoul, Korea, were still lower than those in Japan or China.

Analyzing the characteristics of recent HIV infection has allowed for the identification of vulnerable groups in whom HIV infection is more prevalent. Therefore, HIV prevention measures can be implemented by targeting these vulnerable groups. For instance, both the UK and the US have previously established surveillance systems for recent HIV infections. As consecutive activities to meet indicators of HIV prevention goals, the UK achieved all UNAIDS 90:90:90 targets in 2018. A total of 4453 new HIV diagnoses in 2018 represented a 32% decline from 2009. This decline was attributed to a rapid decrease in HIV infections in homosexual and other MSM, which is known to be the group with the highest recent HIV infection in the UK^26^. In the US, HIV prevention efforts have succeeded in reducing up to 29.1% of risk behaviors among young MSM, and the availability of pre-exposure prophylaxis has dramatically increased (to 64,763 from 7972 individuals)^27^. According to the US CDC HIV surveillance report (2014–2018), the number of HIV infections and incidence rate decreased compared with those in 2014. As of the end of 2018, the estimated number of HIV infections was 36,400 (38,000 in 2014) and the rate was 13.3 per 100,000 population (14.3 per 100,000 population in 2014)^28,29^.

This study has several limitations. Before this surveillance was established, the total number of specimens tested among patients infected with HIV in the current year were less than 60%, which compares with 2008 (39.0%) and 2009 (59.6%). This data indicates a decreased representation from 2009 and onwards. However, as national statistical data of Korea, it was included and analyzed to elucidate the trend of recent HIV infections.

To reduce and minimize the impact of FRR in the LAg Avidity assay, we collected data on CD4+ T cell count at HIV diagnosis and death from AIDS within one year after the first HIV diagnosis for specimens used in the study. For the CD4+ T cell count at HIV diagnosis, our data were linked to 50.4% (2972/5898) of specimens. If it is assumed that subjects registered without a CD4+ T cell counts (about 50%) are the same as those registered with a CD4+ T cell count, the recent infection would be lower than 20.5%. However, it was not reflected in either the recent or acute infection analysis for characteristics of each variable in this study. It cannot be excluded that some of the excluded cases presenting with less than 200 cells/mm^3^ CD4+ T cell counts at the time HIV diagnosis, or cases who succumbed of AIDS-related complications within 1 year of diagnosis of HIV, may have become infected with HIV within 130 days. Furthermore, we could not obtain data on HIV viral load at HIV diagnosis as recommended by the UNAIDS/WHO^30^. Therefore, some elite controllers and subjects on antiretroviral therapy may be included as FRR. However, we rechecked 1,256 patients with CD4+ T cell count at HIV diagnosis and nine deaths from AIDS within one year after the first HIV diagnosis and reclassified 81 specimens as chronic HIV infection. Moreover, this study was the first to investigate the trend and characteristics of recent HIV infection in the context of increasing newly-detected HIV infection in Korea. In addition, we distinguished the acute HIV infection that was included in the recent HIV infection and analyzed the trend and characteristics of acute HIV infection in this study. This study used approximately 75% specimens from newly-diagnosed HIV cases in Korea could be representative of nationwide recent HIV infection. In contrast, most countries report the rate of recent HIV infection in some large cities or high-risk groups such as MSM, and some studies have been limited to only certain regions of the country^24–26^.

## Conclusion

Since 2012, the number of people in their 20’s has had the highest rate of newly-diagnosed HIV infections in Korea. Recent HIV infections in young individuals were higher than those in older populations. This study has allowed the identification of the characteristics of recent HIV infections among newly-diagnosed cases of HIV infections and of high-risk groups for HIV infection recently in Korea. These results can contribute to national HIV prevention by educating doctors and health care workers about the characteristics that the recent infection is increasing among the people undergoing HIV testing due to disease or suspicion of the infection , and most are found in hospitals and PHCs. These data can be used as an opportunity to educate high risk patients, health care providers, and workers about pre-exposure prophylaxis, and in individuals that test positive for the disease, early HIV treatment at the individual level and at the public health level can be initiated. In addition, the present study could be applied to the development and assessment of national HIV prevention programs, such as promotion strategies (webtoons, websites) targeting high-risk age group of 20’s and for placement of HIV Testing vehicles at specific locations; it could provide valuable data for further HIV research in Korea”.

## Abbreviations

HIV: Human immunodeficiency virus
AIDS: Acquired immunodeficiency disease syndrome
UNAIDS: The United States of America
CDC: The Centers for Disease Control and Prevention
LAg avidity assay: Limiting antigen avidity enzyme immunoassay
ELISA: Enzyme-linked immunosorbent assay
RIHE: Regional Institutes of Health and Environment
KDCA: Korea Disease Control and Prevention Agency
HASNet: The HIV/AIDS Supporting Network System
ODn: Optical density
MDRI: Mean duration of recent infection
US CDC: The United States CDC
FRR: False recent ratio
WHO: World Health Organization
UK: The United Kingdom
MSM: Men who have sex with men
PHC: Public health center
WB: Western blotting
CI: Confidence interval
AOR: Adjusted odds ratio
